# Cost-Effectiveness of Dabigatran Compared to Vitamin-K Antagonists for the Treatment of Deep Venous Thrombosis in the Netherlands Using Real-World Data

**DOI:** 10.1371/journal.pone.0135054

**Published:** 2015-08-04

**Authors:** Merlijn W. J. van Leent, Jelena Stevanović, Frank G. Jansman, Maarten J. Beinema, Jacobus R. B. J. Brouwers, Maarten J. Postma

**Affiliations:** 1 Unit of PharmacoEpidemiology & PharmacoEconomics (PE2), University of Groningen, Groningen, the Netherlands; 2 Deventer Hospital, Deventer, the Netherlands; 3 Unit of Pharmacotherapy & Pharmaceutical Care, University of Groningen, Groningen, the Netherlands; 4 Institute of Science in Healthy Aging & health caRE (SHARE), University Medical Center Groningen, Groningen, the Netherlands; University of Bologna, ITALY

## Abstract

**Background:**

Vitamin-K antagonists (VKAs) present an effective anticoagulant treatment in deep venous thrombosis (DVT). However, the use of VKAs is limited because of the risk of bleeding and the necessity of frequent and long-term laboratory monitoring. Therefore, new oral anticoagulant drugs (NOACs) such as dabigatran, with lower rates of (major) intracranial bleeding compared to VKAs and not requiring monitoring, may be considered.

**Objectives:**

To estimate resource utilization and costs of patients treated with the VKAs acenocoumarol and phenprocoumon, for the indication DVT. Furthermore, a formal cost-effectiveness analysis of dabigatran compared to VKAs for DVT treatment was performed, using these estimates.

**Methods:**

A retrospective observational study design in the thrombotic service of a teaching hospital (Deventer, The Netherlands) was applied to estimate real-world resource utilization and costs of VKA monitoring. A pooled analysis of data from RE-COVER and RE-COVER II on DVT was used to reflect the probabilities for events in the cost-effectiveness model. Dutch costs, utilities and specific data on coagulation monitoring levels were incorporated in the model. Next to the base case analysis, univariate probabilistic sensitivity and scenario analyses were performed.

**Results:**

Real-world resource utilization in the thrombotic service of patients treated with VKA for the indication of DVT consisted of 12.3 measurements of the international normalized ratio (INR), with corresponding INR monitoring costs of €138 for a standardized treatment period of 180 days. In the base case, dabigatran treatment compared to VKAs in a cohort of 1,000 DVT patients resulted in savings of €18,900 (95% uncertainty interval (UI) -95,832, 151,162) and 41 (95% UI -18, 97) quality-adjusted life-years (QALYs) gained calculated from societal perspective. The probability that dabigatran is cost-effective at a conservative willingness-to pay threshold of €20,000 per QALY was 99%. Sensitivity and scenario analyses also indicated cost savings or cost-effectiveness below this same threshold.

**Conclusions:**

Total INR monitoring costs per patient were estimated at minimally €138. Inserting these real-world data into a cost-effectiveness analysis for patients diagnosed with DVT, dabigatran appeared to be a cost-saving alternative to VKAs in the Netherlands in the base case. Cost savings or favorable cost-effectiveness were robust in sensitivity and scenario analyses. Our results warrant confirmation in other settings and locations.

## Introduction

Deep venous thrombosis (DVT) and pulmonary embolism (PE) together are labelled venous thromboembolism (VTE), often characterized by severe disability and impairment of quality of life [1]. Accurate anticoagulant therapy for DVT is important to prevent increase of the blood clot, occurrence of PE, or recurrent DVT [2]. Patients with DVT are recommended to receive direct working low-molecular-weight heparins (LMWHs) for at least five days combined with subsequent administration of vitamin K antagonists (VKAs; e.g. warfarin, acenocoumarol or phenprocoumon) for three to six months [3,4]. In exceptional cases, long-term anticoagulant treatment prolonged to up to twelve months is preferred [5], but grossly, an averaged half year of treatment seems a reasonable postulate.

The optimal effectiveness and safety of VKAs is established in a narrow therapeutic range, with the international normalized ratio (INR) being used internationally as a standardised measure of VKA’s biologic effect [4,6]. To maintain the dose at adequate levels, frequent and long-term laboratory monitoring and dose-adjustment is required [5]. Furthermore, VKA treatment may be complicated with multiple drug and food interactions and with pharmacogenetic variability [7,8].

Overcoming some of these disadvantages of VKAs for both the patient and healthcare provider, new oral anticoagulants (NOACs) have been introduced in the last few years [9]. NOACs can modulate the coagulation cascade without requiring laboratory testing or dose-adjustments because of a predictable pharmacokinetic profile [10]. They can also be administered at a fixed dose and have a rapid onset of action [11].

Dabigatran is a NOAC recently approved by the EMA for the treatment of DVT in Europe [12,13]. In the RE-COVER and RE-COVER II trials, 6 months of treatment with dabigatran 150 mg (twice daily) after initial parenteral anticoagulation was found non-inferior to dose-adjusted VKAs in the treatment of DVT with less major, non-major and minor bleedings in patients using dabigatran [14,15]. Currently, NOACs are not yet included in the Dutch Reimbursement System for the DVT indication. For inclusion, a thorough pharmacoeconomic analysis is one of the requirements set by the Ministry of Health.

The first aim of this study is to estimate resource utilization related to INR monitoring, as these are yet unknown for DVT treatment in the Dutch setting. This estimate will subsequently be translated into INR monitoring costs of patients treated with VKAs. Original real-world data are gathered from the thrombotic service from the Deventer Hospital. Secondly, a cost-effectiveness analysis of dabigatran compared to VKAs for the indication of DVT will be performed, using data from the pooled RE-COVER and RE-COVER II trial, combined with the Deventer Hospital data.

## Methods

### Ethics statement

The study was conducted in accordance with the requirements of the Dutch act on protection of personal data [16]. Only anonymous data were used. Observational research with data does not fall within the ambit of the Dutch Act on research involving human subjects and does not require the approval of an ethics review board. This study conformed to the principles embodied in the Declaration of Helsinki [17].

### Real-world data gathered from Deventer Hospital

To estimate resource utilization and translate this into INR monitoring costs, a retrospective observational study design was used. All patients treated in the year 2013 with VKAs for the indication of DVT at the thrombotic service from Deventer Hospital (a Dutch teaching hospital) were enrolled, except those few patients who switched to self-measurement during this year. The latter and the limitation to DVT was done to achieve a sizeable and homogeneous DVT-only study population. Indeed, both initial PE and self-measurement were scarce during our observation period in the Deventer Hospital and sizes would not allow reliable estimates for these subgroups. From the available 2013 data, the total treatment period and the following four types of resource utilization were calculated: total of INR measurements, measurements sampled at the thrombotic service, measurements sampled at home and numbers of required dose-adjustments. Means and standard deviations (SD) of all types of resource utilization were calculated. Total resource utilization was divided by total person-years of treatment and subsequently standardized to 180 days (in the base case). In addition, resource utilizations were translated into INR monitoring costs by multiplying INR measurements sampled at the thrombotic service by €10.37 and multiplying INR measurements sampled at home by €12.77 [18].

### Decision model

The retrospective observational study design from Deventer Hospital and data from the pooled RE-COVER and RE-COVER II trials were used as the basis to calculate the cost-effectiveness of dabigatran compared to VKAs as anticoagulant therapy for the indication DVT [19]. The trials were double-blind, double-dummy, randomized trials, comparing 6 months of treatment with dabigatran at a fixed dose of 150 mg twice daily (dabigatran arm) with 6 months of dose-adjusted warfarin therapy (VKA arm), after initial parenteral anticoagulation with LMWHs. Notably and plausibly, for the purpose of this study warfarin is considered equal to other VKAs, based on the grossly similar efficacy and safety profiles, with acenocoumarol and phenprocoumon specifically being used in the Netherlands [20,21].

**Fig 1:**
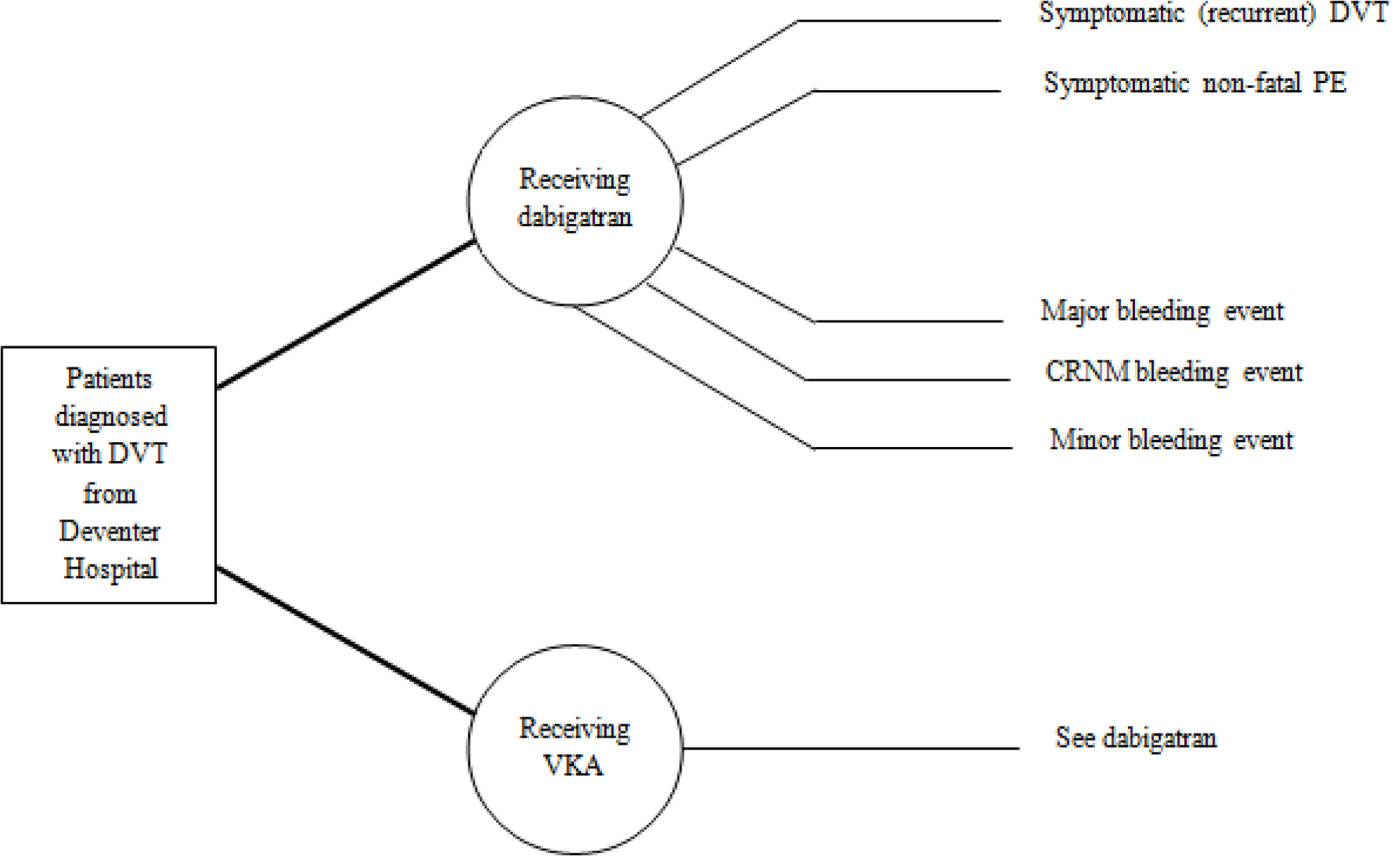
Decision model for the DVT population. The model starts with the diagnosis deep venous thrombosis. All patients directly started with the treatment of VKA or dabigatran anticoagulation, probabilities for events differ per arm. VTE: venous thromboembolism; DVT: deep venous thrombosis; PE: pulmonary embolism; CRNM: clinically relevant non-major.

Following health states were included in the model: symptomatic (recurrent) DVT and symptomatic non-fatal PE, major bleedings, clinically relevant non-major bleedings and minor bleeding events (Fig 1) [22]. To consistently apply conservative assumptions for dabigatran, potential benefits of dabigatran treatment on deaths related to VTE were excluded in this study. Notably, due to the short time horizon of the model, all-cause mortality was also neglected. Relevant probabilities were calculated from corresponding data derived from the pooled RE-COVER and RE-COVER II trials (S1 Table)[19]. Probabilities of patients being in a particular health state were calculated for a treatment period of six months, corresponding to 180 days anticoagulation therapy in the base case. In the base case all differences were evaluated, including all their respective uncertainties within a probabilistic sensitivity analysis.

### Utilities

For every health state and event derived from data from the pooled RE-COVER and RE-COVER II trials, utilities were determined. Utilities represent preferences for a given health state scaled from 0 to 1, where 0 represents death and 1 represents perfect health [23]. Utilities were derived from the literature (S2 Table) [13,24–26] and corrected for the age-specific utility of the specific population targeted (on average 55 year according to the clinical trials) and for the duration in the following way:

$$U_{HSP}=U_{HS}*\;U_{P}$$ (3)

$$TU=(\frac{TDHS}{180}*\;U_{HSP})+(\frac{180-TDHS}{180}*\;U_{P})$$ (4)

U_HSP_ = Utility of a specific health state corrected for the overall age-specific utility of the population concerned U_HS_ = Utility of a specific health state U_P_ = Age-specific utility of the overall population concerned TDHS = Total duration of specific health state within the total treatment period of 180 days TU = Total utility in model during 180 days for a 55-year old DVT-patient

These formulas were applied to each health state (HS) and subsequently summed over all patients in the cohort. Total utility (TU) was interpreted as reflecting the quality adjusted life years (QALYs; duration times utility). QALYs were estimated for the intervention of dabigatran and for the comparator of VKAs. Taking the difference resulted in QALYs gained, which was subsequently inserted in the denominator of the cost-effectiveness ratio (see below).

### Costs

Prices of dabigatran, defined as price per defined daily dose (2x 150 mg) and VKAs were taken from the official Dutch price list (Z-index). Costs of VKA treatment was estimated as a weighted average cost of acenocoumarol and phenprocumon based on their use in the Netherlands (80%:20% respectively) [27,28]. Costs associated with clinical events (VTE, recurrent DVT, PE and major bleeding) were based on actual costs or standard unit costs from the Dutch Cost Manual [29,30]. Minor bleeding costs were estimated as equalling the costs of a general practitioner’s visit and clinical relevant non-major bleeding costs were derived from minor bleeding costs with productivity losses’ costs added [18,31]. INR monitoring costs gathered from data of the thrombotic service of Deventer Hospital were added to the VKA treatment costs, as well as traveling costs and costs due to productivity losses. All prices were expressed in €’s, adjusted to reflect 180 days of treatment and updated to 2013 using the Dutch inflation index (S3 Table) [18,29,31,32].

### Cost-effectiveness analysis

The incremental cost-effectiveness ratio (ICER) was defined as the additional costs of the more expensive treatment divided by its added clinical benefit in QALYs. Whether a therapy is cost-effective and subsequently may be eligible to be included in the Dutch Reimbursement System depends on how much society is willing to pay for a life year gained in perfect health. Informally, a ceiling ratio of €20,000–80,000 per QALY has been suggested in the Netherlands in this respect [33]. Notably, €20,000.00 per QALY can be conceived as maximally strict and very conservative in our analysis. Base case, sensitivity and scenario analyses followed a cohort of 1,000 patients diagnosed with DVT to calculate the effects and costs of VKAs and dabigatran, respectively, and corresponding ICERs.

### Sensitivity & scenario analyses

Univariate sensitivity analyses were conducted in order to inspect the effects of uncertainty in key input parameters and assumptions on the ICER, incremental costs and incremental effects of the base case analysis. Furthermore, to incorporate the uncertainty around all base case parameters simultaneously in the cost-effectiveness analysis a probabilistic sensitivity analysis (PSA) was performed. Univariate sensitivity analysis was conducted by changing the parameters to lower and upper bound values corresponding to 95%- confidence limits from the distributions used in the PSA. Notably, beta distributions for probabilities and utilities and gamma distribution for costs were used in the PSA [34]. PSA was performed by taking a random sample from the assumed input parameter distributions, calculating QALYs, costs and ICERs in 1,000 replications. Results from univariate sensitivity analyses were presented diagrammatically in the form of tornado diagrams, and results from the PSA were plotted on a CE plane and transformed into CE-acceptability curves (CEACs).

Lastly, several scenario analyses were conducted in order to investigate the impact of different factors on the outcome. The first scenario analysis reflected the analysis from the more limited healthcare perspective, whereby production losses and out-of-pocket expenses like travel and parking costs were excluded. Scenario 2 reflected half of production losses in the base case assumed. Furthermore, a third scenario assumed that the treatment period of patients diagnosed with DVT is exactly that found in our observational data for those patients starting and ending treatment in the year of observation (2013) as compared to the 180 days from literature and summaries of product characteristics (SCPs). Scenario 4 calculated the ICER taking only clinically relevant non-major bleedings and minor bleedings into account, as only these were found statistically significantly differing in the pooled analysis. Lastly, scenario 5 calculated the ICER for a treatment period of 90 days to reflect cost-effectiveness for the subgroup with relatively short duration of anticoagulation.

## Results

### Resource utilization

Resource utilization associated with the use of VKAs in The Netherlands, as well as the INR monitoring costs related to the resource utilization are presented in Table 1 for the base case and selected scenarios with differentiating treatment periods. Table 1 shows that the total mean amount of INR measurements was 12.3 (95% CI 11.3, 13.3), 8.1 (95% CI 7.0, 9.2) measured at the thrombotic service and 4.2 (95% CI 3.5, 4.9) measured at home. Reflecting the most conservative duration of treatment in scenario 3, the mean duration of anticoagulation therapy was observed to consist of 143 days, with indeed sizeable subgroups of patients clustered around 180 days and 90 days. During these mean 143 days there was a total mean amount of 15.7 (95% CI 14.4, 17.0) INR measurements (12.0 (95% CI 9.9, 14.1) were measured at the thrombotic service and 3.7 (95% CI 2.8, 4.6) were measured at home). The percentage of INR measurements sampled at the thrombotic service was 66% versus 76%, in the base case and scenario 3, respectively. The INR monitoring costs for the base case analysis and scenario 3 are €138 and €168, respectively.

**Table 1:** Resource utilization and costs of INR monitoring.

	Base case	Scenario 3
Total number of patients	189	46
Mean age	65 (± 16)	56 (± 18)
Mean follow up time (days)	220	143
Treatment period (days)	180	143
INR measurements	12.3 (± 6.7)	15.7 (± 4.5)
Thrombotic service	8.1 (± 8.0)	12.0 (± 7.1)
Home	4.2 (± 5.0)	3.7 (± 3.0)
Dose-adjustments	2.9 (± 4.4)	5.5 (± 3.6)
INR monitoring costs (euros)	138 (α = 3.52, β = 39.17)	168 (α = 52.48, β = 10.25)

The total amount of INR measurements, number of times blood sampling for the measurements was at the thrombotic service, number of times of which blood sampling for the measurements was at home and number of times a dose-adjustment was needed. Means ± standard deviation are shown. The INR monitoring costs follow a gamma distribution and are presented as mean values with shape (α) and rate (β) parameters given in the parenthesis. Base case included all patients treated in the year 2013 with VKAs for the indication of DVT at the thrombotic service from Deventer Hospital. Scenario 3 included only patients who started and stopped treatment in the year 2013. Mean treatment period of these patients was 143 days.

INR, international normalized ratio.

### Cost-effectiveness analysis

For a cohort of 1,000 patients, the total number of events associated with the use of VKAs and dabigatran, as well as the related costs are presented in Table 2. In the base case analysis, dabigatran treatment is associated with an additional QALY of 41.0 (95% uncertainty interval (UI) -18, 97) compared to VKA treatment. Furthermore, VKA treatment involved with additional costs compared to dabigatran, related to INR monitoring costs, travelling costs and costs due to productivity loss. The overall anticoagulant treatment costs were higher for VKA compared to dabigatran (€591,400 versus €572,400), i.e. cost savings of €18,900 (95% UI €-95,832, €151,162) were estimated in the base case analysis (Table 3).

**Table 2:** Events and costs (€, 2013).

	VKA		Dabigatran	
	Number of events	Costs (€)	Number of events	Costs (€)
**Efficacy**				
Symptomatic (recurrent) DVT^1^ ^,^ ^2^	16.6	26,000	12.5	19,500
Symptomatic (recurrent) non-fatal PE^1^ ^,^ ^2^	4.6	22,800	10.3	51,300
**Safety**				
Major bleeding*^3^	18,9	94,000	15.7	78,000
Non-major or CRNM bleeding^3^	79.1^4^	7,900	55.7^1^	5,590
Minor bleeding^3^	193.6^4^	5,000	156.6^1^	4,070
**Other costs**				
Costs of anticoagulants	**-**	14,800	-	414,000
Costs of INR monitoring	**-**	137,500	-	-
Travel costs	**-**	78,500	-	-
Costs due to productivity loss	**-**	205,000	-	-
**Total costs**				
Societal perspective^5^	**-**	591,400	-	572,500
Healthcare perspective	**-**	308,000	-	572,500

Total number of events of symptomatic (recurrent) DVT, symptomatic (recurrent) non-fatal PE, bleeding complications and related costs from societal perspective within a hypothetical patient population of 1,000 subjects receiving dabigatran and VKA for 180 days (base case analysis).

VKA: vitamin-K antagonist; VTE: venous thromboembolism; DVT: deep venous thrombosis; PE: pulmonary embolism: CRNM: clinically relevant non-major; INR, international normalized ratio.

^1^Until the end of the post-treatment period

^2^First occurrence of primary efficacy endpoint

^3^During double-dummy period

^4^Statistically significant differences between VKA and dabigatran

^5^Productivity losses by those 50% of the patients being 64 years-of-age and younger.

**Table 3:** Incremental costs, QALYs and ICER for patients with DVT receiving 180 days anticoagulation therapy from the societal perspective (base case analysis) for a cohort of 1000 patients.

Treatment	Costs (€)	QALYs	Δ Cost	Δ QALY	ICER (€/QALY)
**VKA**	€ 591,400	5,351	€-18,900	41.0	Cost saving
**Dabigatran**	€ 572,400	5,392			

QALY: quality adjusted life year; DVT: deep venous thrombosis; ICER: incremental cost-effectiveness ratio; VKA: vitamin-K antagonist.

### Sensitivity analysis

**Fig 2:**
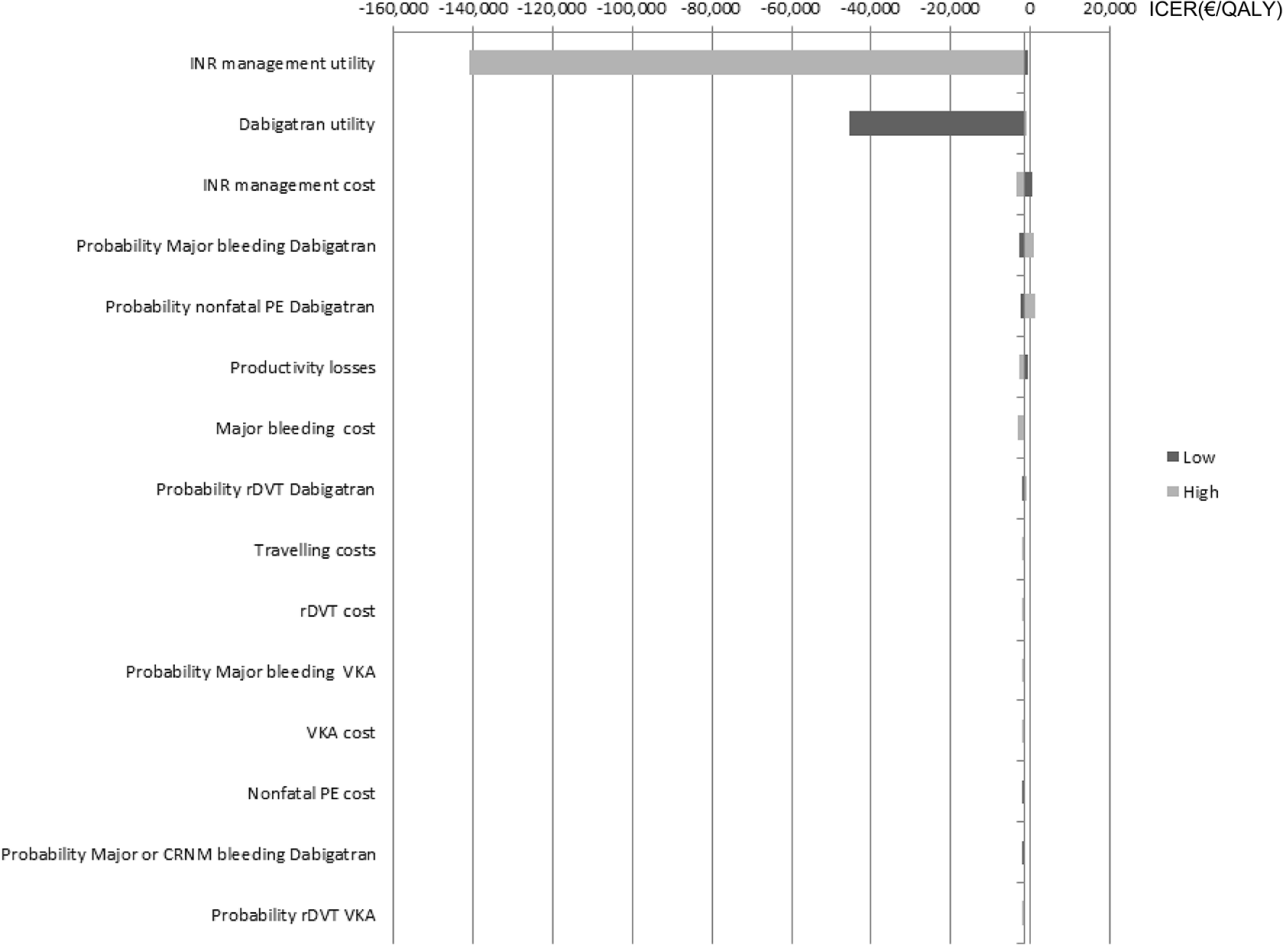
Tornado diagram illustrating the impact on the ICER from sensitivity analyses for dabigatran vs. vitamin-K antagonists. Light grey bars denote influence of the high value of the 95% confidence interval range and dark grey bars denote influence of the low value for parameters investigated. The solid vertical line represents the base case incremental costs per QALY for dabigatran compared to VKA. Horizontal bars indicate the range of incremental costs per QALY obtained by setting each variable to the values shown while holding all other values constant. ICER, incremental cost-effectiveness ratio; INR, international normalized ratio; PE, pulmonary embolism; rDVT, recurrent deep vein thrombosis; VKA, vitamin K antagonist; CRNM, clinically relevant non-major.

**Fig 3:**
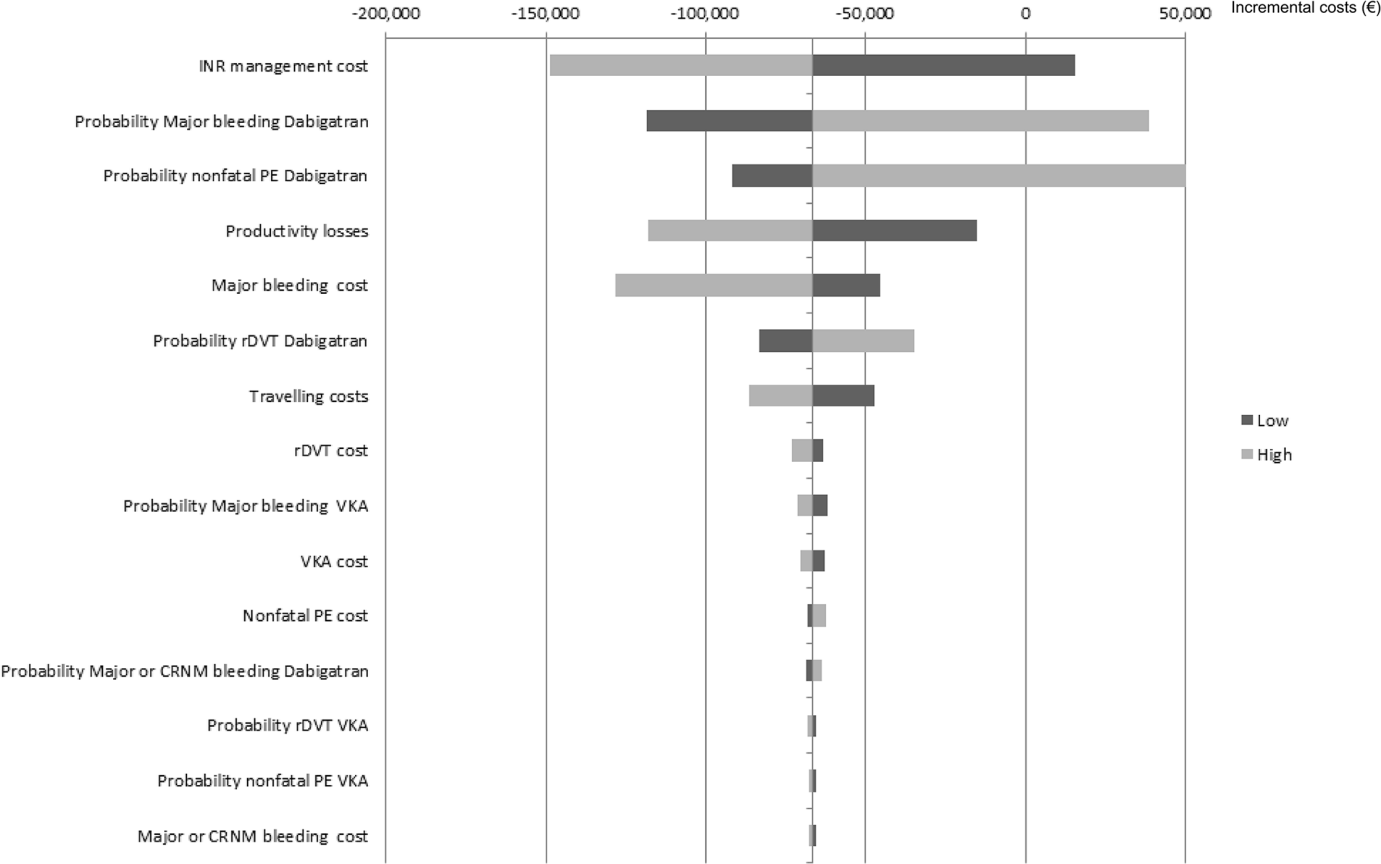
Tornado diagram illustrating the impact on the incremental costs from sensitivity analyses for dabigatran vs. vitamin-K antagonists. Light grey bars denote influence of the high value of the 95% confidence interval range and dark grey bars denote influence of the low value for parameters investigated. The solid vertical line represents the base case incremental costs for dabigatran compared to VKA. Horizontal bars indicate the range of incremental costs obtained by setting each variable to the values shown while holding all other values constant. INR, international normalized ratio; PE, pulmonary embolism; rDVT, recurrent deep vein thrombosis; VKA, vitamin K antagonist; CRNM, clinically relevant non-major.

**Fig 4:**
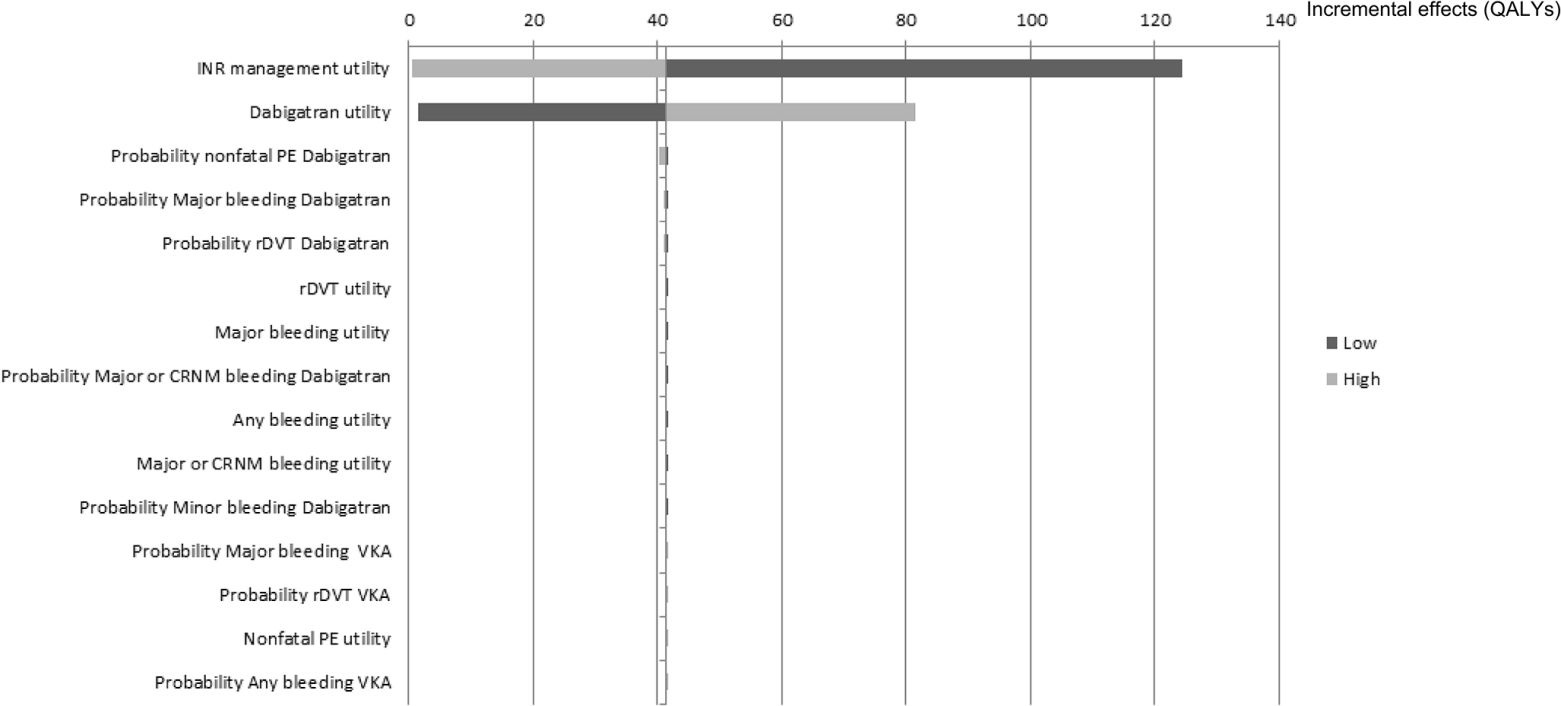
Tornado diagram illustrating the impact on the incremental effects from sensitivity analyses for dabigatran vs. vitamin-K antagonists. Light grey bars denote influence of the high value of the 95% confidence interval range and dark grey bars denote influence of the low value for parameters investigated. The solid vertical line represents the base case incremental QALYs for dabigatran compared to VKA. Horizontal bars indicate the range of incremental QALYs obtained by setting each variable to the values shown while holding all other values constant. INR, international normalized ratio; PE, pulmonary embolism; rDVT, recurrent deep vein thrombosis; VKA, vitamin K antagonist; CRNM, clinically relevant non-major.

**Fig 5:**
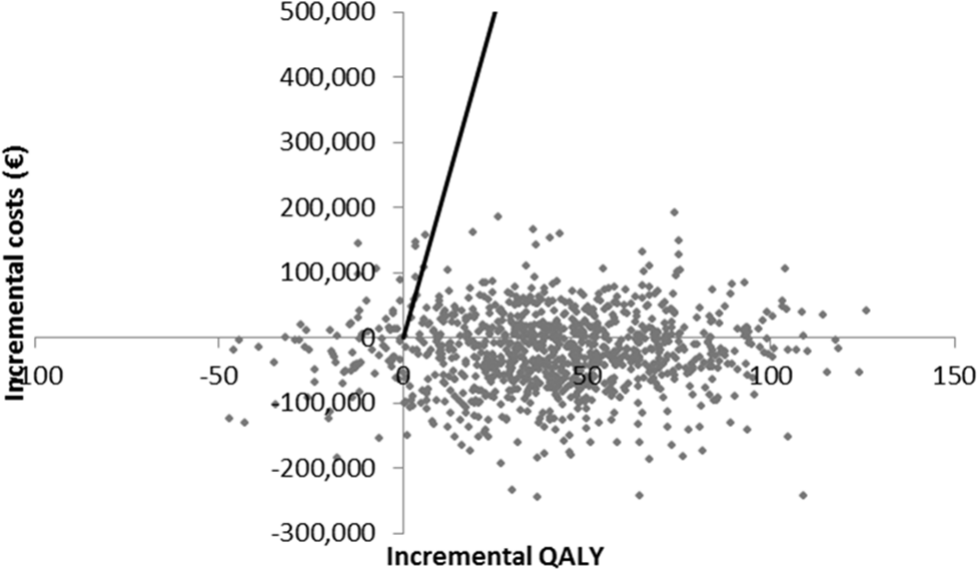
Cost-effectiveness plane in the base case analysis. The graph shows the results of the probabilistic sensitivity analysis of dabigatran treatment compared to VKA treatment in DVT over a period of 180 days from the societal perspective. Points below the diagonal line represent simulations in which dabigatran was a cost-effective alternative at a threshold of €20,000/QALY, below the x-axis, cost-saving points are shown.

**Fig 6:**
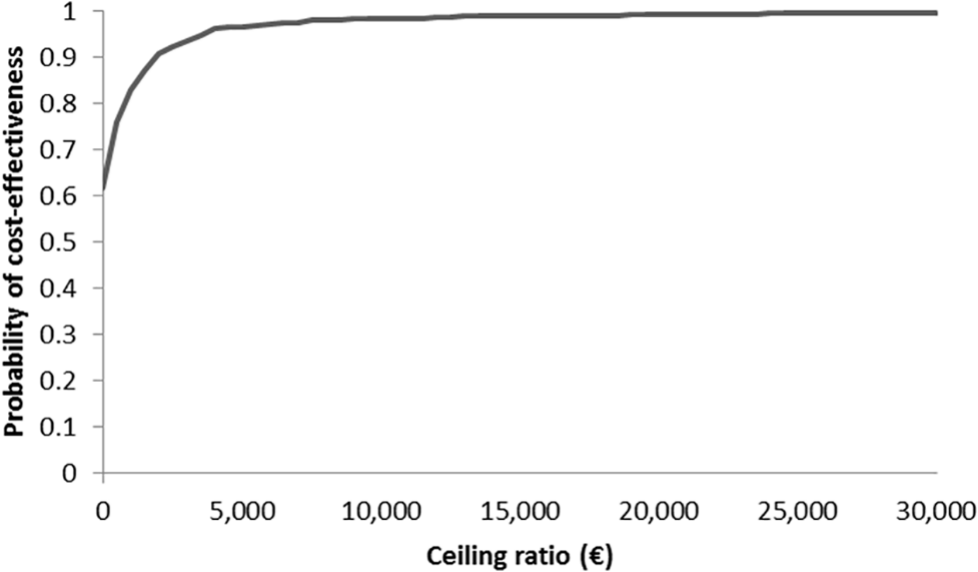
Cost-effectiveness acceptability curve in the base case analysis.

The results of univariate sensitivity analyses for the top 15 parameters by the order of influence they have to the ICER, incremental costs and effects are presented in the form of tornado diagrams (Figs 2–4). The uncertainty around the level of utility associated with INR monitoring, and with dabigatran use, price of INR monitoring, the price of dabigatran, probabilities of major bleeding and nonfatal PE with dabigatran and, the costs due to productivity loss showed the highest impact on uncertainty in the estimated ICERs, incremental costs and effects. Results from the PSA were plotted in the cost-effectiveness plane (Fig 5) and transformed into a CEAC. The CEAC of the base case analysis shows that dabigatran is a cost-effective alternative compared to VKA at a willingness-to-pay threshold of €20,000/QALY in 99% of the simulations and cost saving in 74% of simulations (Fig 6).

### Scenario analyses

The impact of different factors on the ICER was further investigated through scenario analyses (Table 4). From the healthcare perspective, dabigatran is associated with additional QALYs of 41.0 and additional costs of €264,400 compared to VKA. This results in a potentially cost-effective treatment of dabigatran compared to VKA with an ICER at €6,450 per QALY gained (scenario 1). Taking only half of production losses into account results in additional costs of €83,600 for dabigatran compared to VKA. Dabigatran may be considered cost-effective at €2,038per QALY gained (scenario 2). For the conservative DVT treatment duration of 143 days (scenario 3), cost savings remained. When only taking the statistically significant differences into account during 180 days anticoagulant therapy from the societal perspective (scenario 4) results in marginal additional QALYs and still slight cost savings for dabigatran compared to VKA. Finally, for the DVT treatment duration of 90 days (scenario 5), cost savings are shown.

**Table 4:** Incremental costs, QALYs and ICER in various scenarios.

Treatment		Costs (€)	QALYs	Δ Cost	Δ QALY	ICER (€/QALY)
**Scenario 1**	**VKA**	€308,000	5,351	€264,400	41.0	€6,450
	**Dabigatran**	€572,400	5,392			
**Scenario 2**	**VKA**	€488,800	5,351	€83,600	41.0	€2,038
	**Dabigatran**	€572,400	5,392			
**Scenario 3**	**VKA**	€613,100	5,351	€-125,700	41.0	Cost saving
	**Dabigatran**	€487,300	5,392			
**Scenario 4**	**VKA**	€ 13,000	1,797	€-3,300	0.1	Cost saving
	**Dabigatran**	€ 9,700	1,797			
**Scenario 5**	**VKA**	€ 442,000	5,349	€-7,700	41.0	Cost saving
	**Dabigatran**	€ 365,000	5,390			

Patients diagnosed with DVT receiving 180 days anticoagulation therapy from the healthcare perspective (scenario 1), receiving 180 days anticoagulation therapy with half of production losses (scenario 2), receiving 143 days anticoagulation therapy from the societal perspective (scenario 3), only taking statistically significant differences into account during 180 days anticoagulant therapy from the societal perspective (scenario 4), and receiving 90 days anticoagulation therapy from the societal perspective (scenario 5), all for a cohort of 1000 patients.

QALY: quality adjusted life year; DVT: deep venous thrombosis; ICER: incremental cost-effectiveness ratio; VKA: vitamin-K antagonist.

## Discussion

Dabigatran compared to VKA for the treatment of DVT was estimated a cost-saving alternative with €18,900 saved and an additional 41.0 QALYs gained in a cohort of 1,000 DVT patients in our study’s base case analysis from the societal perspective. The economic benefits observed with dabigatran treatment correspond with a generally better safety profile (i.e. less non-major or CRNM and minor bleeding events), a lower number of recurrent DVT events and their associated costs compared to VKA, but also an absence of productivity loss costs associated with INR monitoring. QALYs gain with dabigatran compared to VKA can be largely attributed to disutility associated with INR monitoring in treatment with VKA vs. general population level of utility attached to treatment with dabigatran,

Here, cost savings or favorable cost-effectiveness were robust in scenario and sensitivity analyses. The base case ICER was found to be below the Dutch informal threshold of €20,000/QALY in 99% of PSA simulations. Yet, comparing dabigatran treatment with VKA from the healthcare perspective resulted in the highest ICER found in our study at €6,450/QALY, due to the productivity loss costs and traveling costs which were excluded. In this situation, dabigatran is still cost-effective considering the informal Dutch threshold of €20,000/QALY. Comparing 143 days (average from real-life data) of dabigatran treatment with VKA from the societal perspective resulted in higher cost savings compared to the base case 180 days of treatment.

Despite the cost-savings estimated in our base case compared to VKA treatment, dabigatran treatment may be associated with some disadvantages. During dabigatran treatment, the contacts with the thrombotic service are assumed to disappear and medication surveillance and monitoring will be limited. As a result, early detection of an inadequate anticoagulant level might decrease, while concerns on side-effects and therapeutic benefits may compromise compliance [35]. Still, some studies suggest that INR monitoring during VKA treatment not necessarily improves adherence [36,37]. Yet, caution is warranted in interpreting our results and confirmation in other settings and locations inside and outside the Netherlands is warranted. Notably, similar analyses for the other NOACs are relevant as well in this respect to support findings for the NOACs as a group.

Our cost-effectiveness analysis has several limitations. Firstly, one relates to the time in therapeutic range (TTR). We assumed that the efficacies taken from the pooled trials, which were based on target INR ranges of 2.0 to 3.0 (TTR in most countries), are applicable to the Netherlands (with a target INR range of 2.0 to 3.5). Next, the efficacies of dabigatran and VKAs are based on pooled trials with a follow-up of 6 months, so that long-term risks of adverse events after the 6 months cannot be established and incorporated in the analysis. Notably, the ‘real life’ long-term benefits of the use of NOACs still need to be proven. Thirdly, in the model, no difference in the initial treatment with LMWHs was assumed for the respective analyses of VKAs and dabigatran. From the thrombotic service of Deventer Hospital it could be identified that the mean treatment period of parenteral anticoagulation was 12.7 days with a mean of 4 INR measurements in combination with VKA in contrast to dabigatran which has a mean treatment period of down to 5 days of parenteral anticoagulation [15]. Also, VKAs have a 10 times higher risk of drug and food interactions compared to NOACs. Therefore, there might be patients having to switch from VKA to the more expensive NOACs, which was not included in the model [38]. Ergo, motivations for both under- and over-estimation of ICERs exist. Finally, we mention that between the Dutch government and the manufacturer a price-volume agreement exists in which by increasing volume of dabigatran sales the price reduces. This means that potentially cost savings may increase and/or ICERs may improve further due to this agreement.

Furthermore, recent insights into the development and approval of dabigatran have raised questions about the safety and effectiveness of dabigatran without monitoring and dose adjustment [39]. This has led to a discussion about whether patients taking dabigatran should not also need INR monitoring and plasma-level testing to reduce the risk of bleeding or clotting and ensure maximum benefit from the drug [40,41]. Obviously, this will influence our cost-effectiveness analysis, where the absence of monitoring is a crucial part of the model. Still, all pharmacoeconomic models for dabigatran so far have this absence of monitoring as core building block in the analysis, inclusive a recent analysis for NICE [42]. This analysis in the United Kingdom shows an ICER of £18,200/QALY, still cost-effective according to British norms. The difference with our estimates may be explained amongst others by differences in country-specific costs and the third-party payer perspective taken, excluding productivity loss and traveling costs [42]. Finally, we note that our analysis is specific for DVT; whereas other studies, inclusive the NICE-analysis, take the composite of VTE.

Our model has generalities as well as specificities. We did not subdivide the resource utilization of patients treated with acenocoumarol compared to patients treated with phenprocoumon. Patients on phenprocoumon have more often INR values in the TTR and require less intensive resource utilization compared to patients on acenocoumarol. This is particularly relevant for longtime treatment as it takes a relatively long time before patients reach the optimal INR value of 2.0 to 3.5 on phenprocoumon [8]. Also, before calculating the resource utilization of patients diagnosed with DVT from the Deventer Hospital, patients on self-measurement were excluded. These patients were scarce in Deventer. Including large numbers of these patients though would result in increased cost savings for dabigatran compared to VKA because of the high costs of self-measurement. Finally, we have focused on DVT only—excluding initial PE–to be specific. Further work should be directed to a similar analysis for initial PE, inclusive real-life data gathering. Notably, our results are conditional on the assumptions made as well as the specific data gathered in the Deventer Hospital. The transformation of our results to other settings should be considered with great care as INR monitoring is organised quite differently across countries.

We conclude that we have designed the first study using real-world observational data of this specific kind in the Netherlands. These data appeared highly useful in our cost-effectiveness analysis. Cost savings appeared in the base case, univariate sensitivity and various scenario analyses. In scenarios lacking cost savings, dabigatran was estimated highly cost-effective. The favorable pharmacoeconomic profile was robust in probabilistic sensitivity analysis. Confirmation of our findings in other settings, localities and for other NOACs is warranted to support our study outcomes.

## Supporting Information

S1 Table Probabilities (%) applied in the model. Probabilities were calculated from the 6 months pooled RE-COVER and RE-COVER II study (base case analysis) and were assumed to follow a beta-distribution in the PSA. VTE: venous thromboembolism; DVT: deep venous thrombosis; PE: pulmonary embolism; CRNM: clinically relevant non-major.
(DOCX)

S2 Table Utilities applied in the model. Utilities are determined for every health state. These utilities are not corrected for the norm population and treatment period and were assumed to follow a beta-distribution in the PSA. VTE: venous thromboembolism; DVT: deep venous thrombosis; PE: pulmonary embolism; CRNM: clinically relevant non-major.
(DOCX)

S3 Table Cost parameters applied in the model (€, 2013). Costs are determined for every health state and were assumed to follow gamma-distributions in the PSA. Same events costs are used for the scenario analysis. Anticoagulant costs and other costs are corrected to reflect the base case analysis (180 days) and specific scenario analysis (143 days). VTE: venous thromboembolism; DVT: deep venous thrombosis; PE: pulmonary embolism; CRNM: clinically relevant non-major; ^1^major bleeding utility is based on gastro-intestinal bleeding; ^2^cost estimates that were only available as single point estimates were assumed to follow a gamma distribution with a 25% standard deviation of the mean.
(DOCX)
