# Supplementary material for: Cost-Effectiveness of Dabigatran Compared to Vitamin-K Antagonists for the Treatment of Deep Venous Thrombosis in the Netherlands Using Real-World Data
Source: PLoS One. 2015 Aug 4;10(8):e0135054. doi: 10.1371/journal.pone.0135054 (PMC4524689; doi:10.1371/journal.pone.0135054)
Supplement: S1 Table — Probabilities were calculated from the 6 months pooled RE-COVER and RE-COVER II study (base case analysis) and were assumed to follow a beta-distribution in the PSA. VTE: venous thromboembolism; DVT: deep venous thrombosis; PE: pulmonary embolism; CRNM: clinically relevant non-major. (DOCX) [file pone.0135054.s001.docx]

**S1 Table Probabilities (%) applied in the model.**

| Parameter | Mean | Range |
| --- | --- | --- |
| VTE related death  Warfarin  Dabigatran  (recurrent) DVT  Warfarin  Dabigatran  (non-fatal) PE  Warfarin  Dabigatran  Major bleeding event  Warfarin  Dabigatran  Major or CRNM bleeding  Warfarin  Dabigatran  Minor bleeding  Warfarin  Dabigatran | 0.0  0.0  1.7  2.0  0.5  0.5  1.9  1.2  7.9  4.3  19.4  13.6 | -  0.8 – 2.0  0.4 – 2.9  0.4 – 1.1  0.4 – 0.7  0.6 – 0.8 |

Probabilities were calculated from the 6 months pooled RE-COVER and RE-COVER II study (base case analysis) and were assumed to follow a beta-distribution in the PSA.

VTE: venous thromboembolism; DVT: deep venous thrombosis; PE: pulmonary embolism; CRNM: clinically relevant non-major
