# Supplementary material for: Cost-Effectiveness of Dabigatran Compared to Vitamin-K Antagonists for the Treatment of Deep Venous Thrombosis in the Netherlands Using Real-World Data
Source: PLoS One. 2015 Aug 4;10(8):e0135054. doi: 10.1371/journal.pone.0135054 (PMC4524689; doi:10.1371/journal.pone.0135054)
Supplement: S2 Table — Utilities are determined for every health state. These utilities are not corrected for the norm population and treatment period and were assumed to follow a beta-distribution in the PSA. VTE: venous thromboembolism; DVT: deep venous thrombosis; PE: pulmonary embolism; CRNM: clinically relevant non-major. (DOCX) [file pone.0135054.s002.docx]

**S2 Table Utilities applied in the model.**

| Parameter | Mean | Range | Reference |
| --- | --- | --- | --- |
| Norm population | 0.9 | 0.86 – 0.94 | [24] |
| (recurrent) DVT (1 month) | 0.8 | 0.64 – 0.98 | [26] |
| (non-fatal) PE (1 month) | 0.6 | 0.36 – 0.86 | [26] |
| Major bleeding (2 weeks) | 0.7 | 0.49 – 0.86 | [25] |
| CRNM bleeding (2 days) | 0.7 | 0.49 – 0.86 | [25] |
| Minor bleeding (5 days) | 0.9 | 0.89 – 1.00 | [13] |
| INR monitoring | 1.0 | 0.86 – 0.99 | [25] |
| Dabigatran | 1.0 | 0.96 – 1.00 | [26] |

Utilities are determined for every health state. These utilities are not corrected for the norm population and treatment period and were assumed to follow a beta-distribution in the PSA.

VTE: venous thromboembolism; DVT: deep venous thrombosis; PE: pulmonary embolism; CRNM: clinically relevant non-major
