# Supplementary material for: Cost-Effectiveness of Dabigatran Compared to Vitamin-K Antagonists for the Treatment of Deep Venous Thrombosis in the Netherlands Using Real-World Data
Source: PLoS One. 2015 Aug 4;10(8):e0135054. doi: 10.1371/journal.pone.0135054 (PMC4524689; doi:10.1371/journal.pone.0135054)
Supplement: S3 Table — Costs are determined for every health state and were assumed to follow gamma-distributions in the PSA. Same events costs are used for the scenario analysis. Anticoagulant costs and other costs are corrected to reflect the base case analysis (180 days) and specific scenario analysis (143 days). VTE: venous thromboembolism; DVT: deep venous thrombosis; PE: pulmonary embolism; CRNM: clinically relevant non-major; 1major bleeding utility is based on gastro-intestinal bleeding; 2cost estimates that were only available as single point estimates were assumed to follow a gamma distribution with a 25% standard deviation of the mean. (DOCX) [file pone.0135054.s003.docx]

**S3 Table Cost parameters applied in the model (€, 2013).**

| Parameter | Mean | Range | Reference |
| --- | --- | --- | --- |
| Costs of events |  |  |  |
| (recurrent) DVT | 1,562 | 781 – 3,125 | [29] |
| (non-fatal) PE | 4,976 | 2,486 – 9,952 | [29] |
| Major bleeding | 4,977 | 1,995 –13,588^1^ | [29] |
| Non-major or CRNM | 100 | 75 – 125^2^ | [18] |
| Minor bleeding | 26 | Fixed | [18] |
| Costs of anticoagulants |  |  |  |
| VKA | 14.76 | 11.07 – 18.45^2^ | [28] |
| Dabigatran | 414.00 | fixed | [27] |
| Other costs |  |  |  |
| INR monitoring | 137.51 | 55.45 – 219.55 | Calculated |
| Travelling costs | 78.35 | 58.77 – 97.94^2^ | [31] |
| Productivity loss costs | 410.03 | 307.53 – 512.53^2^ | [31] |

Costs are determined for every health state and were assumed to follow gamma-distributions in the PSA. Same events costs are used for the scenario analysis. Anticoagulant costs and other costs are corrected to reflect the base case analysis (180 days) and specific scenario analysis (143 days).

VTE: venous thromboembolism; DVT: deep venous thrombosis; PE: pulmonary embolism; CRNM: clinically relevant non-major; ^1^major bleeding utility is based on gastro-intestinal bleeding; ^2^cost estimates that were only available as single point estimates were assumed to follow a gamma distribution with a 25% standard deviation of the mean.
